# Phenome-wide association study using research participants’ self-reported data provides insight into the Th17 and IL-17 pathway

**DOI:** 10.1371/journal.pone.0186405

**Published:** 2017-11-01

**Authors:** Margaret G. Ehm, Jennifer L. Aponte, Mathias N. Chiano, Laura M. Yerges-Armstrong, Toby Johnson, Jonathan N. Barker, Suzanne F. Cook, Akanksha Gupta, David A. Hinds, Li Li, Matthew R. Nelson, Michael A. Simpson, Chao Tian, Linda C. McCarthy, Deepak K. Rajpal, Dawn M. Waterworth

**Affiliations:** 1 Target Sciences, GlaxoSmithKline, Collegeville, PA, United States of America; 2 Genomic Medicine, PAREXEL International, Durham, NC, United States of America; 3 Target Sciences, GlaxoSmithKline, Stevenage, United Kingdom; 4 Division of Genetics and Molecular Medicine, Faculty of Life Sciences & Medicine, Kings College London, London, United Kingdom; 5 Suzanne F Cook Epidemiology Associates LLC, Chapel Hill, NC, United States of America; 6 Dermatology, GlaxoSmithKline, Research Triangle Park, NC, United States of America; 7 Statistical Genetics, 23andMe, Inc., Mountain View, CA, United States of America; Universite de Montreal, CANADA

## Abstract

A phenome-wide association study of variants in genes in the Th17 and IL-17 pathway was performed using self-reported phenotypes and genetic data from 521,000 research participants of 23andMe. Results replicated known associations with similar effect sizes for autoimmune traits illustrating self-reported traits can be a surrogate for clinically assessed conditions. Novel associations controlling for a false discovery rate of 5% included the association of the variant encoding p.Ile684Ser in *TYK2* with increased risk of tonsillectomy, strep throat occurrences and teen acne, the variant encoding p.Arg381Gln in *IL23R* with a decrease in dandruff frequency, the variant encoding p.Asp10Asn in *TRAF3IP2* with risk of male-pattern balding, and the *RORC* regulatory variant (rs4845604) with protection from allergies. This approach enabled rapid assessment of association with a wide variety of traits and investigation of traits with limited reported associations to overlay meaningful phenotypic context on the range of conditions being considered for drugs targeting this pathway.

## Introduction

T helper (Th) 17 cells produce the cytokine interleukin 17 (IL-17) and play a role in multiple autoimmune and infectious diseases. There are promising drugs, including several anti IL-17 or anti IL-17 receptor (*IL-17R*) antibodies, targeting the Th17 and IL-17 pathway for dermatology and autoimmune diseases, some of which have been recently approved and others that are in development [1]. The anti IL-17 antibodies were efficacious for psoriasis but seemed to increase both disease activity and adverse events including infection when investigated in patients with active Crohn’s disease [CD] [2, 3]. A study to identify novel associations for genes in this pathway has the potential to suggest new indications and highlight possible safety concerns for existing and emerging drugs [4]. This is particularly useful in the drug development context since genetically supported targets are demonstrated to have increased success rates in clinical development [5].

In the last 10 years, the genetic contribution to common disease has been investigated by performing increasingly large genome wide association studies (GWAS), whereby polymorphisms along all chromosomes are tested for association with a trait of interest. GWAS results have provided a growing catalog of the genetic loci that play a role in disease pathogenesis. Identifying causal variants is difficult and usually more work is required to identify the mechanism by which a variant influences a clinical trait [6]. An alternate approach which can provide insight into possible biological disease mechanisms is a phenome-wide association study (PheWAS), whereby a particular variant is studied for association with a wide variety of traits to profile the phenotypic effects of a gene variant. This approach is relevant to drug discovery when a variant is known to have a functional effect and the available traits include conditions which have not been previously studied, since there is focus on anticipating the effects of modulating the gene products as drug targets. Denny et al., [7] applied the PheWAS paradigm using electronic medical records (EMRs) to study the relationships between genetic variants and multiple traits in 13,835 participants. In the present study, we used the approach with a much larger database (521,000) of self-reported health histories from research participants of the genetics company, 23andMe, to investigate the relationship between variants in genes from the Th17 and IL-17 pathway to provide insight into the functional consequences of modulating the proteins encoded by these genes.

We selected seven variants from seven genes known to be in the Th17 and IL-17 pathway, for PheWAS analysis. We included variants with established GWAS associations and/or those predicted to have functional consequences. We evaluated associations across the selected gene variants to look for common patterns of association. Selected variants were also compared with previous association reports for autoimmune diseases, to evaluate how genetic analysis of these self-reported phenotypes compare to more traditional case-control study results. Results for two of the variants (encoding p.Arg381Gln in *IL23R* and p.Ile684Ser in *TYK2*) which were included in a PheWAS of 1,358 EMR-derived traits evaluated in 13,835 European-ancestry individuals from five sites of the Electronic Medical Records and Genomics (eMERGE) network (7) were compared with the results from this study. Lastly, PheWAS results from each variant were more comprehensively assessed, evaluating each gene from a drug target perspective. While this study focused on only 7 genes within the pathway, the results provide insight into the use of PheWAS in drug discovery research.

## Results

### Variant selection and association analyses

We evaluated variants in 57 genes (Wikipathways www.wikipathways.org) from the Th17 and IL-17 pathway, denoted IL-17 signaling pathway in homo sapiens (S1 Table) and identified seven variants either known or predicted to be functional, or associated with multiple common conditions with minor allele frequencies (MAF) greater than 0.5%. S2 Table lists the seven variants along with associations previously reported for each variant [5]. Genotype data for these seven variants were available from genome wide array genotyping data and any missing values were imputed. Each variant was tested for association with 1254 health-related traits, derived from self-reported phenotype data. A total of 521,000 study participants with greater than 97% European ancestry (determined through analysis of local ancestry) with phenotype data for the questions of interest were included. Analyses were adjusted for age, gender, and the top five ancestry principal components to account for residual population structure. The false discovery rate (FDR) was controlled at 5% over analyses for all 7 variants [8]. We report FDR *q*-values throughout the paper, except when compared to previously reported *p*-values. Power was estimated across a variety of traits (with widely differing sample sizes) for the MAFs observed for all variants to assist with interpretation of results.

## Association results

**Fig 1:**
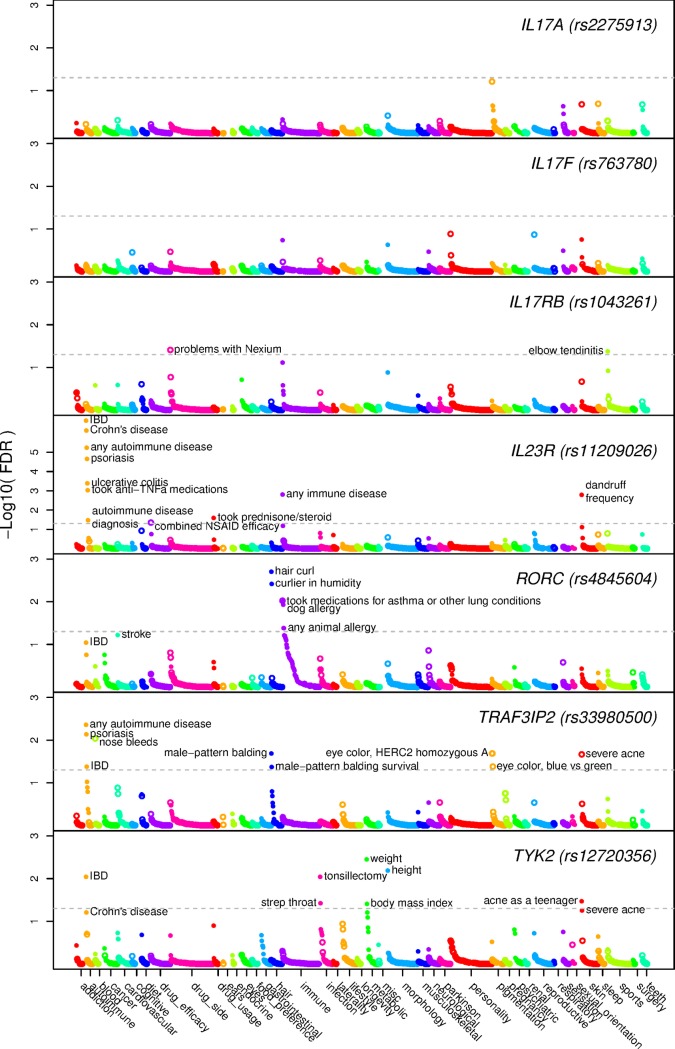
PheWAS results for variants in seven genes. False Discovery Rate q-values for 1254 traits are plotted for each variant evaluated. Closed circles depict association of the minor allele with increased risk of the conditions and open circles with protection from the conditions. Horizontal dashed lines indicate the q = 0.05 FDR threshold.

**Fig 2:**
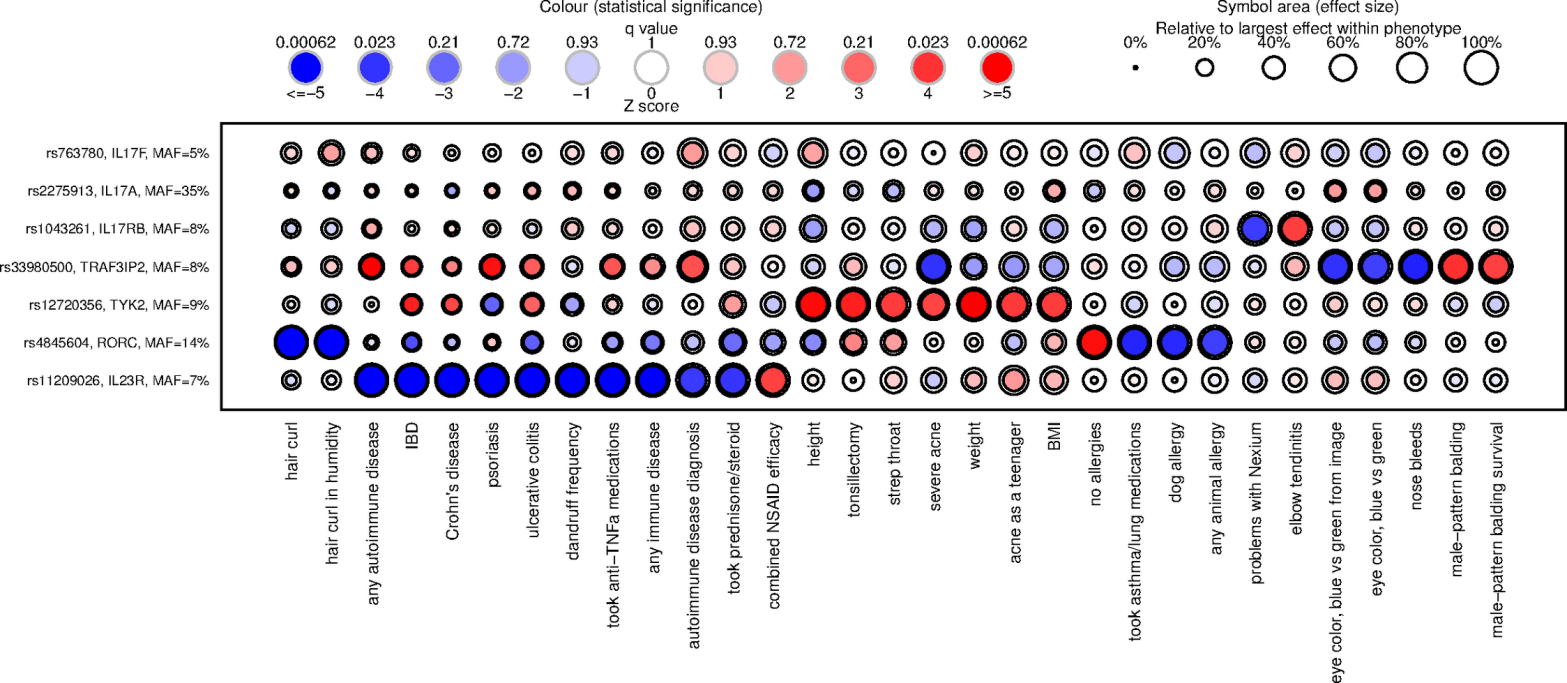
Heat map of selected PheWAS associations. Associations are shown for each variant with each trait, for the set of all traits that had association with any -variant at 5% FDR. For each association, black circles are drawn with area proportional to the effect size point estimate (inner circle) and 95% upper confidence limit (outer circle), scaled relative to the maximum within each trait. The inner circle is colored according to the effect direction (blue to red) and significance (color scale according to association Z score, with corresponding q value cut-offs shown in the legend). Traits and [variants] are ordered using hierarchical clustering on Euclidean distances between columns [and rows] of the association Z score matrix.

Association results for all seven variants with all 1254 traits are shown in Fig 1, S3 Table lists all results significant at 5% FDR and S4 Table lists all results. For four variants, the *IL23R* missense variant encoding p.Arg381Gln (rs11209026; c.1142G>A; MAF = 0.07), the *RORC* regulatory variant (rs4845604; c.70+225C>T; MAF = 0.14), the *TRAF3IP2* missense variant encoding p.Asp10Asn (rs33980500; c.28G>A; MAF = 0.08), and the *TYK2* missense variant encoding p.Ile684Ser (rs12720356; c.2051T>G; MAF = 0.09), we observed a large number of highly significant associations (six or more per variant at 5% FDR), including traits with both previously known and novel associations. Fig 2 shows a heat map of association statistics, for all traits associated with at least one variant at 5% FDR. A cluster of autoimmune traits (ulcerative colitis, psoriasis, inflammatory bowel disease [IBD]—Crohn’s disease or ulcerative colitis) have associations for variants in *IL23R* and *TRAF3IP2* genes. Beyond the associations with autoimmune traits, the variants in *IL23R*, *RORC*, *TRAF3IP2* and *TYK2* seem to have variant specific, rather than pathway-related, association patterns. For these four variants, and also for the *IL17RB* nonsense variant encoding p.Gln484Stop (rs1043261; c.1450C>T; MAF = 0.08), we identified, at 5% FDR, a number of previously unreported associations, albeit some at borderline significance levels, which we discuss further below. We did not observe any significant associations for the *IL17A* promoter variant (rs2275913; c.-197G>A; MAF = 0.35) or the *IL17F* missense variant encoding p.His161Arg *(*rs763780; c482A>G; MAF = 0.05). These negative results are consistent with the relatively weaker *a priori* evidence for either large functional effects or phenotypic associations for these two variants, but are also consistent with a possible functional effect but lower power for the *IL17F* variant encoding p.His161Arg due to the lower MAF. Fig 2 shows that, for some traits, the *IL17F* variant encoding p.Asp10Asn may have comparable effect sizes to other variants evaluated here (shown by size of the circles), although at lower statistical significance levels (shown by color intensity). These results provide evidence that the *IL17A* variant we selected is unlikely to have a large effect on the human traits studied (upper bounds shown by outer circle size in Fig 2), likely due to insufficient functional impact. Overall, these results suggest that genes involved in the Th17 and IL-17 pathway have variable and heterogeneous mechanistic influences on a range of human disease phenotypes.

### Comparison to GWAS results

**Fig 3:**
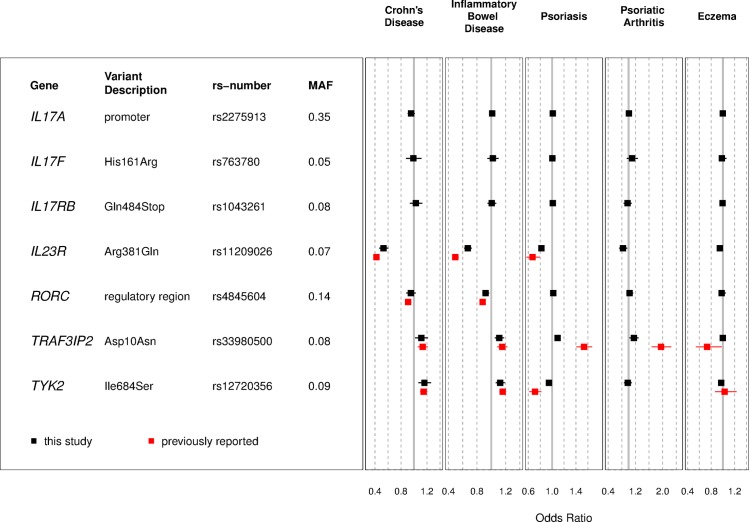
Association and literature results. Summary of association statistics for study results and previously reported associations for Crohn’s disease, IBD, psoriasis, psoriatic arthritis and eczema.

We investigated similarities and differences of the current PheWAS results, with association results available in the literature that were based on clinician assessment or validated diagnostic instruments. Fig 3 and S5 Table compare the association statistics for Crohn’s disease, IBD, psoriasis, psoriatic arthritis and eczema. Of fourteen previously reported associations summarized in Fig 3, nine were previously associated with genome-wide significance levels (*p*≤5.0×10^−8^). For all fourteen of the previously reported associations, the odds ratios for this dataset were in the same direction as the previously reported associations although we observed significantly different (*p*<0.05) effect estimates for 7 of the 14 associations (S6 Table). These differing estimates could be attributed to factors such as differences in sample size, phenotype definitions, patient demographics or to the winner’s curse [9].

### Power analyses

For PheWAS studies, power will be highly variable due to the varying sample sizes across phenotypes, precision of the phenotype and allele frequency of the variant. To support interpretation, we performed a comprehensive power analysis of traits, for the given allele frequencies. S7 Table summarizes results from an analysis of power across a variety of traits including the minimum odds ratio (OR) or effect size (β), in response standard deviations per allele, for binary or continuous traits, respectively, for each variant where there is 80% power to identify an additive effect assuming the given allele frequency, sample size and α = 0.0002, the p-value threshold which empirically corresponds to the FDR cut-off of 5%. No adjustment to account for precision of the phenotype was made. For the variants studied, there is 80% power to identify small effects (OR>1.15; β>0.06) for 26–71% of the traits across the MAF range, moderate effects (OR>1.5; β>0.11) for 87–95% of the traits and large effects (OR>2; β>0.15) for nearly 90% of the traits (S8 Table). Review of the power results by trait category highlights that there is 80% power to detect moderate (OR>1.5; β>0.11) or large effects (OR>2; β>0.15) for more than 75% of the traits within all trait categories except drug side effects, drug efficacy and Parkinson’s disease related traits. However, there is 80% power to detect small effects (OR>1.15; β>0.06) for 65% of the traits only within the trait categories of cognition, sleep, infection, lifestyle, sensation, longevity and personality.

### Interpretation of results by variant

The *IL23R* gene is involved in Th17 regulation and regulates IL-17 production [10]. The IL17-induced Th17 effector function is impaired in the presence of rs11209026 encoding p.Arg381Gln. This minor allele encoding the glycine residue has also been previously associated with twofold protection against psoriasis and threefold protection against Crohn’s disease [10]. Consistent with previous reports, in this study, the minor allele was associated with protection from Crohn’s disease (OR = 0.53; *q* = 6.63x10^-28^), IBD (OR = 0.67; *q* = 8.0x10^-31^), psoriasis (OR = 0.82; *q* = 7.64x10^-20^), and a composite of 25 auto-immune conditions (OR = 0.88; *q* = 9.37x10^-20^). The minor allele was also associated with traits likely driven by these disease associations: in participants reported as taking anti-TNFα medications (OR = 0.72; *q* = 3.92x10^-8^) and in patients reported as taking prednisone or other steroid medications (OR = 0.95; *q* = 0.026). We also observed a novel and highly significant association with a decrease in dandruff frequency (β = -0.08; *q* = 6.61x10^-7^). This is unlikely to be driven by other trait associations since dandruff frequency was not significantly correlated with the other associated traits (S1 Fig). The variant was included in a previously reported PheWAS analysis which failed to identify any statistically significant associations using a Bonferroni threshold of p<0.05/1358 = 3.7x10^-5^ [7].

The RORγT transcript of the *RORC* gene is an important transcription factor in Th17 differentiation. The rs4845604 variant is hypothesized to have a regulatory effect on expression and may be associated with reduced levels of the transcript. This variant was the only common variant in *RORC* with a previously reported genetic association–with IBD [11]. The *q*-value for association of the rs4845604 with IBD was *q* = 0.18 which failed to meet the *q*<0.05 threshold. We observed an association with the minor allele (A) of this variant with decreased hair curl (β = -0.09; *q* = 9.92x10^-31^). The variant is 261kb from rs17646946 (MAF = 0.24; *r*^*2*^ = 0.02 and *D’* = 0.20 with rs4845604) within the *TCHH* gene, which has been associated with hair curl in multiple studies [12, 13]. The Eriksson *et al* paper [12] studied participants that were included in this PheWAS. The association of rs4845604 with hair curl is likely to be novel, but further conditional analyses would be required to determine if this signal and previously report associations implicating *TCHH* may tag the same underlying genetic effect. We observed associations of the minor allele with protection from multiple allergies including dog (OR = 0.92; *q* = 0.019) and any animal (OR = 0.95; *q* = 0.042), and with subjects receiving medications for asthma or other lung conditions (OR = 0.95; *q* = 0.008). Additionally, there were associations with *q*-values just above the 0.05 threshold with protection from mold allergy (OR = 0.95; *q* = 0.062), any food allergy (OR = 0.94; *q* = 0.069) and grasses allergy (OR = 0.96; *q* = 0.072). The minor allele was not associated with protection from asthma (OR = 0.97; *q* = 0.22). The associated traits showed high levels of correlation between different allergy traits and between allergy and the asthma related traits (S1 Fig).

*TRAF3IP2* encodes ACT1, a signaling adaptor involved in the regulation of adaptive immunity. The variant, rs33980500, encoding p.Asp10Asn has been shown to prevent ACT1 from interacting with TRAF6, suggesting that this variant may modulate downstream signals of different crucial immunoreceptors through altered TRAF interactions [14]. The variant had been previously reported as associated with IBD [11, 15], psoriasis [16], and psoriatic arthritis [14]. Only psoriasis (OR = 1.09; *q* = 0.0019) and IBD (OR = 1.11; *q* = 0.042) were found to have a significant association in this dataset compared to previously reported odds ratios (OR = 1.53; 95% CI [1.41,1.67]) for psoriasis [16] and (OR = 1.15; 95% CI [1.09,1.22]) for IBD [15]. Despite previous reports of an association with psoriatic arthritis (OR = 1.95; *p* = 1.13x10^-20^) [14], and 80% power to detect an OR of 1.29 in this dataset, the association with psoriatic arthritis is non-significant. Significant associations were observed for protection from frequent nose bleeds (OR = 0.87; *q* = 0.0063) and severe acne (OR = 0.93; *q* = 0.022), for green eye color as measured on a blue versus green scale (β = -0.04; *q* = 0.026), for risk of male pattern balding before age 40 (OR = 1.17; *q* = 0.021) and male pattern balding (analyzed using survival analysis—Hazard Ratio = 1.09; *q* = 0.042). Further evaluation of the association of this variant and acne vulgaris in 1893 cases and 5132 controls (imputation r^2^ = 0.99 with p = 0.51) did not replicate the observed results [17, 18]. A look-up in an association analysis of male pattern balding in CoLaus including 578 cases and 547 controls demonstrated that this variant was associated with male pattern balding (OR = 1.34, *p* = 0.02; 95% CI of [1.05, 1.72]) with imputation r^2^ of 0.91 [19]. Review of a published GWAS using 23andMe database for blue versus green eye color (31,037 cases and 30,741 controls) identified association with the G allele of rs13200059 (OR = 1.26; 95% CI [1.18, 1.35]; *p* = 2.7x10^-11^). The credible set for the region includes the *TRAF3IP2* gene. The effector gene in this locus is more likely to be *SLC16A10* which is an aromatic amino acid transporter where the drosophila orthologue of this gene, *KAR*, is known to be involved in visual pigmentation (33). Review of Pearson correlation coefficients between associated traits (S1 Fig) highlighted correlation within the related male pattern baldness traits and within the related eye color traits but not between the trait groupings suggesting that these associations may represent true pleiotropy.

*TYK2* is a member of the Janus kinase (JAK) family of proteins that mediates signaling downstream of several cytokine receptors and intercellular adhesion molecule genes [20]. This gene contains a number of common coding variants including the missense variants rs34536443, encoding p.Pro1104Ala, and rs12720356 encoding p.Ile684Ser. We selected the rs12720356 variant for study as it was the only missense variant previously predicted to be deleterious with MAF greater than 5% with previous disease association reports: Crohn’s disease [11] and psoriasis [21]. A recent analysis of the functional impact of variants in the *TYK2* gene established that minor allele homozygosity at the rs34536443 SNP drives a near-complete loss of TYK2 function which impairs type I IFN, IL-12 and IL-23 signaling whereas rs12720356 does not have similar biological effects [22]. Analysis of publicly available eQTL data suggests that the rs1270356 or the rs5030390 variant that is in linkage disequilibrium with it, may have an effect on ICAM1 expression in stimulated CD14+ monocytes suggesting the *ICAM1* gene which may explain why rs34536443 confers protection from multiple diseases while more heterogeneous effects were noted for rs1270356 [22]. We observed significant associations between this variant and increased weight (β = 0.88; *q* = 4.10x10^-5^) and height (β = 0.06; *q* = 0.0011) as well as associations with risk of tonsillectomy (OR = 1.06; *q* = 0.0061), IBD (OR = 1.12; *q* = 0.0061), more severe acne as a teenager (β = 0.03; *q* = 0.034), strep throat occurrences (β = 0.04; *q* = 0.038), and BMI (β = 0.09; *q* = 0.039). None of these associations were replicated in a PheWAS of 13,835 European-ancestry individuals which included the variant, although the case counts for tonsillitis, IBD and acne related phenotypes (S9 Table) were all less than 262 [7]. The associations with height and BMI were not replicated with N = 119,206 participants (*p* = 0.21) [23] and N = 112,363 (p = 0.17) [24], respectively. Review of both the BMI and height studies failed to identify any variants within 1 Mb of the p.Ile684Ser variant that could explain the association of this variant with height and BMI that we observed. The association with IBD was previously reported (OR = 1.16; *p*-value = 4.3x10^-16^) [11]. In addition to the significant association with risk of more severe acne as a teenager, we observed an association with risk of severe acne with a *q-*value just exceeding the 0.05 threshold (OR = 1.07; *q* = 0.056). This variant was evaluated for association with risk of acne vulgaris in 1893 cases and 5132 controls and was found to be modestly associated (OR = 1.135; 95% CI [1.0048, 1.283]; *p* = 0.045) [17, 18]. An association with protection from psoriasis has been reported in the literature (OR = 0.71; *p* = 4.00x10^-11^) [21] but not found in this dataset (OR = 0.95; *q* = 0.20) despite 80% power to identify a protective association with an OR less than 0.92 (S7 Table). A previous, smaller PheWAS study of rs12720356 in 29,377 European participants for 502 common and 30 clinical traits related to *TYK2* biology did not identify any significant associations [20].

## Discussion

Our PheWAS study of variants in the Th17 and IL-17 pathway rediscovered known and identified novel significant associations which provide insight into conditions that may be affected by modulation of the protein encoded by the gene. We reviewed results for the four variants that appear to have an effect on human clinical traits, to assess possible interpretations of the PheWAS findings [25]. Considering the results for the variant encoding p.Arg381Gln in *IL23R* gene, we noted that Il-23R and Th17 cytokines are down-regulated after successful therapies and there are monoclonal antibodies directed against IL-12/23p40, IL-17A and IL-17RA which are effective in psoriasis [1, 26]. In addition to psoriasis, therapies directed against *IL23* have been tested in other diseases including psoriatic arthritis, multiple sclerosis and Crohn’s diseases [1]. The association with decrease in dandruff frequency is likely to be due to true pleiotropy since Di Meglio *et al* [10] showed that the individuals homozygous for the Arg381Gln allele in *Il23R* gene were strikingly unresponsive to IL-23, with minimal or no IL-17A and IL-17F production suggesting a shared biological mechanism, and we did not observe correlation between the associated traits of dandruff frequency, Crohn’s disease and psoriasis.

The PheWAS results for *RORC* regulatory variant highlighted associations for the minor allele with protection from allergies, a phenotype that has not been comprehensively studied using a whole genome scan approach. Multiple companies have programs targeting *RORC* including Vitae Pharmaceuticals with a phase II program in psoriasis, and Japan Tobacco with a phase I program for autoimmune and allergic asthma (Pharmaprojects: https://citeline.com/products/pharmaprojects/). If rs4845604 is shown to be associated with reduced levels of the RORγT transcript, then a RORC inhibitor may be an effective treatment for diseases for which this variant has a protective effect, such as asthma.

The PheWAS results for the variant encoding p.Ile684Ser in TYK2 (which we noted was working through the *ICAM1* gene) highlighted a known risk association with IBD and a protective association for psoriasis. While there are no marketed drugs targeting *ICAM1*, efalizumab is an integrin alpha-L/beta-2 inhibitor which was approved for the treatment of psoriasis but then withdrawn due to a potential risk to patients of developing progressive multifocal leukoencephalopathy (FDA. Efalizumab withdrawal. 2009 [updated 11 Apr 2011] http://www.fda.gov/drugs/drugsafety/postmarketdrugsafetyinformationforpatientsandproviders/ucm143347.htm). The integrins and their receptors potentially represent a class of targets of interest for inflammatory and immune diseases.

While our study is not a comprehensive evaluation of the Th17 pathway, the results highlighted that the promoter variant, rs2275913, in *IL17A* and variant encoded by p.Gln484STOP in *IL17RB* are unlikely to have a functional impact on the human traits studied since they are either not associated or associated with a small number with good power while the variant encoding p.Arg381Gln in *IL23R*, regulatory variant, rs4845604, in *RORC*, variant encoding p.Asp10Asn in *TRAF3IP2* and variant encoding p.Ile684Ser in *TYK2* do show evidence that they have an impact on human traits with multiple trait associations. The *TRAF3IP2* and *TYK2* genes impact downstream or more distal signaling and we observed both protective and risk associations with variants in these genes. In contrast, the *IL23R* and *RORC* genes have a more direct impact on IL-17 levels and we observed only protective trait associations for these genes. The strategy provides a more holistic view of the impact of gene variation across the pathway that is not as apparent when genes are evaluated one at a time.

Analysis of these data provided insight into the use of self-reported data. These data included traits that have not been previously studied using a genome scan approach such as dandruff frequency, as well as infection traits like strep throat occurrences. The results obtained using the self-reported data are similar to association results reported by cohort studies (which focus on clinically assessed patients). One example is illustrated by the results for the variant encoding p.Asn10Asp in *TRAF3IP2*. While associations with psoriasis and IBD were reported, the results failed to identify a statistically significant association with psoriatic arthritis–reported as *p* = 1.13x10^-20^ with OR = 1.95 [14]. Psoriatic arthritis is relatively uncommon (prevalence is estimated to vary from 0.3% to 1%). In this dataset we observed a higher than expected prevalence of reported cases in the 23andMe community (1.3%) which may be due to customer misreport, use of inconsistent diagnostic criteria compared to past GWAS cohorts (which used the CASPAR and Moll and Wright criteria [14]), and/or differences in disease spectrum in the 23andMe cohort compared to other clinically ascertained cohorts.

In summary, we were able to confirm numerous reported associations and extend our findings of variants in the TH17 pathway. Specifically, we characterized a regulatory variant in *RORC* gene associated with multiple allergy traits and asthma related traits which have not been reported previously. We identified that the Ile684Ser variant within the *TYK2* gene, which may act through the *ICAM1* gene, is associated with many traits including psoriasis, Crohn’s disease, and infections, and may suggest integrins and their receptors as potential drug targets. Additional associations, beyond those reported in the literature, were observed for the variants with previous association reports suggesting this information provides valuable information about the effect of the variant on human traits. This PheWAS study provided a comprehensive profile of human traits associated with each variant evaluated, including skin, infection and allergy traits, which haven’t been comprehensively studied thus far. This PheWAS does have limitations. Important variants or genes may not have been included in this study which was limited to 7 variants in 7 different genes. The analyses were well powered for moderate and large effect sizes greater than 1.5 for nearly 80% of the traits for all variants reported in European participants but samples sizes were too small to provide a meaningful analysis for other ethnicities and traits. The use of PheWAS in the drug discovery context provides an approach for identifying variants most likely to have an observable effect on gene function and context for the direction of effects suggesting whether a gene activator or inhibitor is needed. Furthermore, clinical trials may want to select or eliminate patients carrying variants with an effect on gene function for drug target genes depending on size and direction of the effect. Interpretation of PheWAS study findings can be complicated by nearby genes, as illustrated by the association of *RORC* and hair curl, and variable power for each trait assessed. We hope that the emergence of EMR-linked biobanks with genetic data will facilitate more routine use of the PheWAS screen providing insights into drug development strategies for targeting these genes and pathways.

## Methods and materials

All research participants included in the analyses provided informed consent and answered surveys online according to 23andMe’s human subject’s protocol, which was reviewed and approved by Ethical & Independent Review Services, a private institutional review board (http://www.eandireview.com).

Informed consent is neither written nor oral—it is electronic. 23andMe customers consent to participate in research online and consent is captured electronically. Ethical & Independent Review Services approved this form of informed consent and waived the requirement to obtain signed consent under 45 CFR 46.117(c). Participants have access to all consent documents through their accounts and may change their consent selection at any time.

Samples were genotyped on one of four genotyping platforms. The genotyping platforms, quality control procedures, ancestry determination and imputation protocols have been previously described [27]. Restriction to greater than 97% European ancestry excluded about 25% of otherwise eligible research participants. Minor allele frequencies (MAF) for the variants included in this study for each genotyping platform are listed in S10 Table.

We tested the association with 1254 well-curated phenotypes, which are distributed among different phenotypic categories (e.g., cognitive, autoimmune, psychiatrics, etc). An overview of the trait data collection process has been previously described [12]. Specific definitions for all traits associated with variants with *q*<0.05 FDR are summarized in S1 Appendix. For case control comparisons, we computed association test results by logistic regression. For quantitative traits, association tests were performed by linear regression. For survival traits, we computed association test results by fitting Cox proportional hazards regression. All regression analyses were performed using R version 3.2.2. We typically assumed additive allelic effects and included covariates for age (as determined by participant date of birth), gender, and the top five principal components to account for residual population structure. The association test *P* value we report was computed using a likelihood ratio test.

Power calculations were performed for binary and continuous traits estimating the minimum OR or effect size (β) in response standard deviations per allele to identify associations using logistic or linear regression, respectively, with an additive model assuming the given sample size, 80% power, minor allele frequencies corresponding to the variants selected (0.05–0.35) and β = 0.0002 which empirically corresponds to the FDR cut-off of 5% in these results. For each allele frequency, the proportion of traits (of 1250 binary or continuous traits) with power to identify small OR and effect sizes (OR>1.15; β>0.06), moderate OR and effect sizes (OR>1.5; β>0.11) and large OR and effect sizes (OR>2; β>0.15) is calculated. The OR ranges were determined based on the 25^th^, 50^th^, and 75^th^ quantiles of the ORs and hazard ratios from the GWAS catalog with p-values ≤ 5x10^-8^ for non-pharmacogenetic and non-anthropometric traits [28]. The effect size ranges were selected so that the distribution of effect sizes within the small, moderate and large effect size range was similar to that for the odd ratios. Correlation between traits involved in associations with FDR less than 5% was summarized using the Pearson correlation coefficient. Welch's *t*-test is used to test for consistency of OR estimates using OR estimates, confidence intervals and sample sizes.

## Supporting information

S1 Fig Pearson correlation coefficients for associated traits. Pearson correlation coefficients for traits associated with traits at 5% FDR.
(TIF)

S1 Table Th17 and IL-17 pathway genes. List of genes evaluated as associated with Th17 and IL-17 pathway (Wikipathways https://www.wikipathways.org and pathway knowledge)
(XLSX)

S2 Table Previously reported genetic associations. Variants evaluated and traits reported as associated by combining data from multiple sources including the National Human Genome Research Institute (NHGRI) GWAS Catalog, the tables and supplementary materials of manuscripts archived in the NHGRI GWAS Catalog, and the database of Genotypes and Phenotypes (dbGaP), among others [5].
(XLSX)

S3 Table Study association statistics. Association statistics (trait, description, gene, rs number, p-value, experiment wide *q*-value, number of cases, number of controls, OR and 95% confidence interval, number of participants, effect size and 95% confidence interval) from 23andMe analysis for traits with FDR significance level of 5%. We report OR for binary traits and effect sizes for quantitative traits including some traits that analyzed ordinal numbers.
(XLSX)

S4 Table Study association statistics. Association statistics (trait, description, gene, rs number, p-value, experiment wide *q*-value, number of cases, number of controls, OR and 95% confidence interval, number of participants, effect size and 95% confidence interval) from 23andMe analysis for traits and levels of significance. We report OR for binary traits and effect sizes for quantitative traits including some traits that analyzed ordinal numbers.
(XLSX)

S5 Table Study and literature association results. Summary of association statistics (p-values, OR, 95% confidence intervals and sample sizes) for results from this study and from the literature with references.
(XLSX)

S6 Table Comparison of effect sizes for immune traits. Summary of p-values for Welch’s *t*-test which was used to test for consistency of OR estimates. Ratio of odd ratios and 95% CI is for effect estimates from this study verses previously reported associations.
(XLSX)

S7 Table Power table for each variant and phenotype. Maximum OR and effect size where there is 80% power to identify an effect assuming an additive model, β = 0.0002, and given number of cases and controls or N and given allele frequency.
(XLSX)

S8 Table Power summary for each variant. Summary of the proportion of traits with 80% power to detect small, moderate and large effects for each variant.
(XLSX)

S9 Table Number of cases for eMERGE PheWAS study. The number of cases for phenotypes of interest in a PheWAS [7].
(XLSX)

S10 Table MAF stratified by array. Minor allele frequencies for all variants for each genotyping array.
(XLSX)

S1 Appendix Phenotype definitions. Survey names, survey questions, and logic used to define cases and controls for traits associated with variants with *q*<0.05 FDR.
(PDF)
