# Supplementary figures and images for: Phenome-wide association study using research participants’ self-reported data provides insight into the Th17 and IL-17 pathway
Source: PLoS One. 2017 Nov 1;12(11):e0186405. doi: 10.1371/journal.pone.0186405 (PMC5665418; doi:10.1371/journal.pone.0186405)

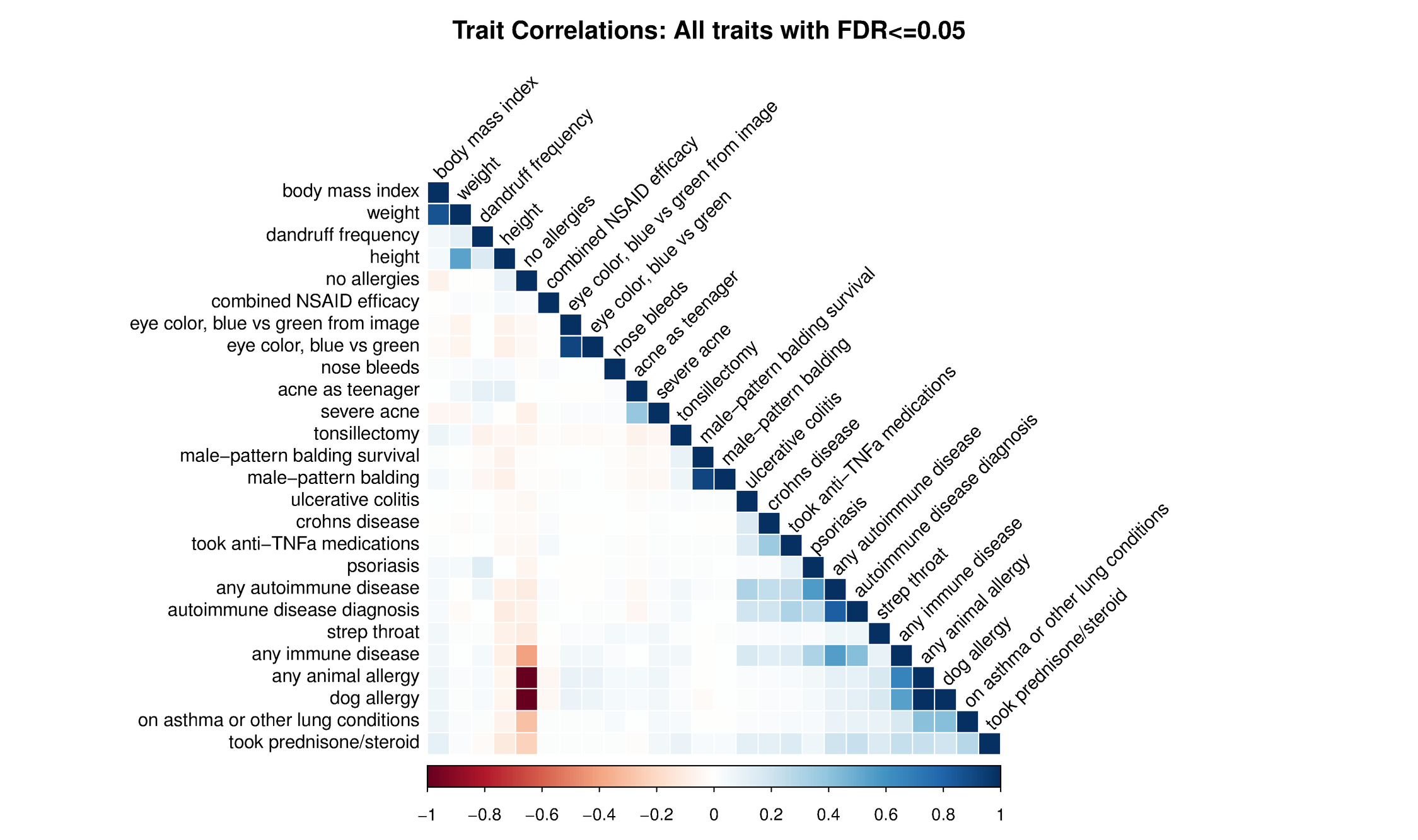

Supplement: S1 Fig — Pearson correlation coefficients for traits associated with traits at 5% FDR. (TIF) [file pone.0186405.s001.tif]
