# Supplementary material for: Phenome-wide association study using research participants’ self-reported data provides insight into the Th17 and IL-17 pathway
Source: PLoS One. 2017 Nov 1;12(11):e0186405. doi: 10.1371/journal.pone.0186405 (PMC5665418; doi:10.1371/journal.pone.0186405)
Supplement: S1 Appendix — Survey names, survey questions, and logic used to define cases and controls for traits associated with variants with q<0.05 FDR. (PDF) [file pone.0186405.s012.pdf]

S1 Appendix

Phenotype definitions: Survey names, survey questions, and logic used to define cases and controls for traits associated with variants with $q<0.05$.

Dog allergy

Dog allergy cases are defined using responses to the following questions in “Allergies and asthma” survey and “Allergies'' survey; Cases first indicated that they had had an allergic reaction to dog, and then reported having either a positive allergy test or at least one of the specified reactions.

Allergies and Asthma Survey:

- Have you had an allergic reaction to any of the following types of animals?
  (Dogs)
- Have you had an allergy test administered by a medical professional, which indicated you were allergic to the following types of animals?
  (Dogs)
- What type of reaction did you have after being exposed to [Dogs]? Please check all that apply.
  - Difficulty swallowing or difficulty speaking
  - Hives (red, itchy, or swollen skin)
  - Itching in your mouth
  - Itchy or runny nose
  - Wheezing or asthma

Allergies Survey:

- Have you ever had an allergic reaction to any of these animals? (Dogs)
- Have you ever had a positive allergy test to any of these animals? (Dogs)
- What type of reaction did you have after being exposed to [Dogs]? Please check all that apply.
  - Difficulty swallowing or difficulty speaking
  - Hives (red, itchy, or swollen skin)
  - Itching in your mouth
  - Itchy or runny nose
  - Wheezing or asthma

We identified controls that reported having no allergies of any kind (see ‘no allergies’ below), based on information from many sources.

any_animal_allergy

Animal allergy cases are defined using responses to the questions in “Allergies and asthma” survey and “Allergies'' survey. Cases first indicated that they had had an allergic reaction to cats, dogs or other animals, and then reported having either a positive allergy test or at least one of the specified reactions.

Allergies and Asthma Survey:

- Have you had an allergic reaction to any of the following types of animals?
  (Cats,Dogs,Another animal)

- Have you had an allergy test administered by a medical professional which indicated you were allergic to the following types of animals?
  (Cats,Dogs,Another animal)
- What type of reaction did you have after being exposed to [Cats,Dogs,Another animal]? Please check all that apply.
  - Difficulty swallowing or difficulty speaking
  - Hives (red, itchy, or swollen skin)
  - Itching in your mouth
  - Itchy or runny nose
  - Wheezing or asthma

Allergies Survey:

- Have you ever had an allergic reaction to any of these animals? (Cats,Dogs,Another animal)
- Have you ever had a positive allergy test to any of these animals? (Cats,Dogs,Another animal)
- What type of reaction did you have after after being exposed to [Cats,Dogs,Another animal]? Please check all that apply.
  - Difficulty swallowing or difficulty speaking
  - Hives (red, itchy, or swollen skin)
  - Itching in your mouth
  - Itchy or runny nose
  - Wheezing or asthma

We identified controls that reported having no allergies of any kind, based on information from many sources. See “no allergies” below.

no_allergies

We identified controls that provide at least one negative response and no positive responses to any of the following definition of allergy.

Your Health Profile survey

- Have you ever been diagnosed by a doctor with any of the following types of allergies?'
  - Seasonal allergies
  - Asthma
  - Environmental (but not seasonal) allergy
  - Food allergy
  - Drug allergy

Asthma Symptoms survey

- Have you ever been diagnosed by a doctor with asthma or bronchial asthma?
- Do you currently have allergies?
- Have you ever had any of the following? Please check all that apply.
  - Positive allergen skin prick test or positive allergen blood test
  - Atopic dermatitis/Eczema (chronic itchy and scaly skin rashes caused by allergies)
  - Allergic rhinitis (stuffed or dripping nose caused by allergies)
  - Allergic conjunctivitis (red, itchy, or painful eyes caused by allergies)

Allergies and Asthma survey and Allergies Survey

- Questions about a total of 38 allergens, including foods, plants, animals, molds, latex, medicines, vaccines, and other
- Have you ever had an asthma attack?

any_autoimmune

The any_autoimmune phenotype combines diagnoses for the following specific disease phenotypes. Cases have at least one positive response to any of these questions. Controls have at least one negative response and no positive responses.

* Rheumatoid arthritis
* Type 1 diabetes
* Psoriasis
* Systemic lupus erythematosus
* Crohn's disease
* Ulcerative colitis
* Multiple sclerosis
* Celiac disease
* Scleroderma
* Autoimmune hepatitis
* Ankylosing spondylitis
* Guillain-Barre syndrome
* Sjogren's syndrome
* Goodpasture syndrome
* Wegener's granulamatosis
* Polymyalgia rheumatica

any_immune

The any_immune phenotype combines diagnoses for the following specific disease phenotypes. Cases have at least one positive response to any of these questions. Controls have at least one negative response and no positive responses.

* Rheumatoid arthritis
* Type 1 diabetes
* Psoriasis
* Systemic lupus erythematosus
* Crohn's disease
* Ulcerative colitis
* Multiple sclerosis
* Celiac disease
* Scleroderma
* Autoimmune hepatitis
* Ankylosing spondylitis
* Guillain-Barre syndrome
* Sjogren's syndrome

* Goodpasture syndrome
* Wegener's granulamatosis
* Polymyalgia rheumatica
* Any Allergy
* Allergic asthma
* Asthma
* Chronic hives
* Eczema

weight

A quantitative phenotype (in pounds) defined using the responses to the question ‘What is your current weight?’ from the following surveys. The first response from these sources is selected. People with age under 18 or missing age are removed. Weight values outside the range of 30 to 800 pounds have been removed.

1. Your Health Habits survey
2. Your Health Profile
3. Health Intake survey
4. The Roots into the Future intake form

height:

Quantitative trait looking at self-reported Height in inches, restricted to the range 48--90 (4" to 7"6'). Data collected from:

1. Your Health Habits survey
2. Your Health Profile
3. Health Intake survey
4. The Roots into the Future intake form

People with age under 18 or missing age are removed. Height values less than 4 feet or greater than 7 feet 6 inches have been removed.

BMI:

The bmi phenotype is computed from self-reported ‘height’ and ‘weight’, which come from the following surveys:

1. Your Health Habits survey
2. Your Health Profile
3. Health Intake survey
4. The Roots into the Future intake form

The first response from these sources is selected. Height values less than 4 feet or greater than 7 feet 6

inches, and weight values outside the range of 30 to 800 pounds have been removed. BMI values outside the range of 5 to 70, and individuals under the age of 18, are also removed. The final phenotype is in units of kg/m^2^.

crohns:

The "crohns" phenotype comes from several sources:

Your Medical History survey:
- Have you ever been diagnosed by a doctor with any of the following intestinal conditions? (Crohn's disease)

A research snippet:
- Have you ever been diagnosed with Crohn's disease?

Your Profile and Health History:
- Have you ever been diagnosed with or treated for any of the following conditions (Crohn's disease)

Health Intake survey (branched version):
- Have you ever been diagnosed or treated for any of the following conditions? (An autoimmune disease)
- What autoimmune diseases have you been diagnosed with? Please check all that apply. (Crohn's disease)
- Have you ever been diagnosed or treated for any of the following conditions? (A stomach, bowel, or other gastrointestinal problem)
- What stomach, bowel, or other gastrointestinal disorders have you been diagnosed with? Please check all that apply. (Crohn's disease)

We combined the questions in 'Health Intake survey (branched version)' to define intermediate cases (when any response to the diagnosis of Crohn’s disease is a yes) and intermediate controls (when it is not a case and at least one response is a no). Individuals with discordant responses were defined as cases.

We then evaluate the first 3 survey questions and the above-derived intermediate phenotype in the listed order, and an individual’s case or control status was determined by their first response.

Health Followup Survey
- In the last 2 years, have you been newly diagnosed with or started treatment for any of the following conditions? (Crohn's disease)

At last, we combined the above-derived response and the responses for the above extra follow up question to define cases (when any response is a yes) and controls (when it is not a case and at least one response is control). Individuals with discordant responses were defined as cases.

ulcerative_colitis

The "ulcerative_colitis" phenotype comes from multiple sources:

Your Medical History survey:

- Have you ever been diagnosed by a doctor with any of the following intestinal conditions? (Ulcerative colitis)

A research snippet:

- Have you ever been diagnosed with ulcerative colitis?

Your Profile and Health History:

- Have you ever been diagnosed with or treated for any of the following conditions

(Ulcerative colitis)

Health Intake survey (branched version):

- Have you ever been diagnosed or treated for any of the following conditions?

(An autoimmune disease)

- What autoimmune diseases have you been diagnosed with? Please check all that apply. (Ulcerative colitis)
- Have you ever been diagnosed or treated for any of the following conditions? (A stomach, bowel, or other gastrointestinal problem)
- What stomach, bowel, or other gastrointestinal disorders have you been diagnosed with? Please check all that apply. (Ulcerative colitis)

We combined the questions in 'Health Intake survey (branched version)' to define intermediate cases (when any response to the diagnosis of ulcerative colitis disease is a yes) and intermediate controls (when it is not a case and at least one response is a no). Individuals with discordant responses were defined as cases.

We then evaluate the first 3 survey questions and the above-derived intermediate phenotype in the listed order, and an individual’s case or control status was determined by their first response.

Health Followup Survey

- In the last 2 years, have you been newly diagnosed with or started treatment for any of the following conditions? (Ulcerative colitis)

We then combined the above-derived response and the responses for the above extra follow up question to define cases (when any response is a yes) and controls (when it is not a case and at least one response is control). Individuals with discordant responses were defined as cases.

crohns_or_ulcerative_colitis

The "crohns_or_ulcerative_colitis" phenotype combines 'crohns' and 'ulcerative_colitis' two phenotypes. Cases reports of either Crohn's disease diagnoses or ulcerative colitis diagnoses. Controls have none of the two diseases.

elbow_tendinitis

Sports Injuries Survey:

- Has a doctor ever diagnosed you with any of the following kinds of tendinitis (tendonosis)? Elbow

tendinitis

Responses coded as: cases: "Yes " ; controls: "No " ; NAs: "I'm not sure "

eye_image_blue_v_green_qt

This is a quantitative phenotype, which comes from combining two questions:

Research Snippets:

- Which best describes your natural eye color? (Blue; Bluish grey; Green and blue; Green and brown; Hazel; Amber (yellowish-brown); Light brown; Dark brown; I'm not sure)

Pigmentation Survey:

- Look very closely at the color of your eyes, preferably under bright natural light. Then complete the following: the image that most closely resembles the color of my eyes is: (7 images, from blue to brown)

Blue eyes are coded 0, greenish_blue color eyes are coded 1, green eyes are coded 2.

eyecolor_blue_v_green

The blue versus green eye color phenotype is derived from combining two questions:

Research Snippets:

- Which best describes your natural eye color? (Blue; Bluish grey; Green and blue; Green and brown; Hazel; Amber (yellowish-brown); Light brown; Dark brown; I'm not sure)

Pigmentation Survey:

- Look very closely at the color of your eyes, preferably under bright natural light. Then complete the following: the image that most closely resembles the color of my eyes is: (7 images, from blue to brown)

People with blue or bluish grey eyes are controls, those with green or greenish-blue are cases.

eyecolor_herc2_blue

The eyecolor phenotype is quantitative trait derived from combining two questions:

Research Snippets:

- Which best describes your natural eye color? (Blue; Bluish grey; Green and blue; Green and brown; Hazel; Amber (yellowish-brown); Light brown; Dark brown; I'm not sure)

Pigmentation Survey:

- Look very closely at the color of your eyes, preferably under bright natural light. Then complete the following: the image that most closely resembles the color of my eyes is: (7 images, from blue to brown)

scored 0=blue to 6=dark brown. The phenotype was further limited to individuals who are homozygous GG at rs12913832 in HERC2.

hair_curl

Quantitative trait looking at hair curl based on data from the following questions:

Pigmentation Survey:

- Is your hair naturally straight or curly? Answers were drawings of degrees of hair curl, coded as straight, slightly_wavy, wavy, big_curls, small_curls, very_tight_curls)

Research Snippets:

- Is your hair naturally straight or curly?" Answers = straight, slight_wave, wavy, big_curls, small_curls, tight_curls)

Answers to the questions above were mapped to levels as below:

* 0 = straight
* 1 = slightly_wavy OR slight_wave
* 2 = wavy
* 3 = big_curls
* 4 = small_curls
* 5 = very_tight_curls OR tight_curls

iqb.acne_as_teenager

Quantitative trait based on the following question:

Research Snippets:

- When you were a teenager, how would you describe the amount of acne you had? (None, Mild, Moderate, Severe, Very severe, I'm not sure, Not applicable)

The first five responses are scored from 0 to 4.

severe_acne

Your Profile and Health History:

- Have you ever been diagnosed with or treated for severe acne (requiring a prescription medication)?
- Have you ever been diagnosed with or treated for any of the following conditions? Severe acne (requiring a prescription medication)"

Health intake Survey (branched version):

- Have you ever been diagnosed or treated for any of the following conditions? A skin condition"
- What skin conditions have you had? Please check all that apply. Severe acne

We combined the last two 'Health Intake survey (branched version)' to define intermediate cases (when any response is a yes) and intermediate controls (when it is not a case and at least one response is control). Individuals with discordant responses were defined as cases.
We then evaluate the above 2 questions and the derived intermediate phenotype in the listed order, and an individual's case or control status was determined by their first response.

iqb.curlier_in_humidity

Research Snippet:

- Does your hair become more wavy or curly in humid weather?" Responses coded as: cases: "Yes"; controls: "No"; NAs: "I'm not sure "

iqb.dandruff_frequency

Research Snippet:

- How often do you have dandruff? (If you use treatments for your dandruff, please answer for the frequency of occurrence without treatment.)
  Responses coded as:

NA: "I'm not sure"
0: "Never or almost never"
1: "Rarely"
2: "Sometimes"
3: "Often"
4: "Always or almost always"

iqb.ever_diagnosed_autoimmune

Cases were defined as answering "Yes"; Controls were defined as answering "No", to the following question. to the question above.

Research Snippet:

- Have you ever been diagnosed with an autoimmune disease (a disease in which your immune system attacks part of your body)?

iqb.nose_bleeds

Research Snippet:

- Do you frequently develop nose bleeds?" Responses coded as: cases: "Yes" ; controls: "No" ; NAs: "I'm not sure"

iqb.strep_throat

Research snippet:

- How many times have you been told by a doctor that you had strep throat?''

(Never, 1-2 times, 3-5 times, More than 5 times, I'm not sure)

The first four responses are assigned scores from 0 to 3.

psoriasis

The "psoriasis" phenotype combines reports of psoriasis diagnoses from several sources:

Your Medical History survey:

- Have you ever been diagnosed by a doctor with any of the following autoimmune conditions? (Psoriasis)''

The Roots into the Future intake form

- Has a doctor ever told you that have any of these skin conditions? Please check all that apply (Psoriasis)

A research snippet:

- Have you ever been diagnosed with psoriasis?''

Health Intake survey (branched version):

- Have you ever been diagnosed or treated for any of the following conditions?

(An autoimmune disease)''

- What autoimmune diseases have you been diagnosed with? Please check all that apply. (Psoriasis)''

Health Intake survey (branched version):

- Have you ever been diagnosed or treated for any of the following conditions?

(A skin conditions)''

- What skin conditions have you had? Please check all that apply. (Psoriasis)''

We combined the questions in 'Health Intake survey (branched version)' to define intermediate cases (when the response to the diagnosis of psoriasis is a yes) and intermediate controls (when it is not a case and at least one response is a no). Individuals with discordant responses were defined as cases.

We then evaluate the first 3 survey questions and the above-derived intermediate phenotype in the listed order, and an individual’s case or control status was determined by their first response.

Health Followup Survey

- In the last 2 years, have you been newly diagnosed with or started treatment for any of the following conditions? (Psoriasis)

We then combined the above-derived response and the responses for the above extra follow up question to define cases (when any response is a yes) and controls (when it is not a case and at least one response is control). Individuals with discordant responses were defined as cases.

iqb.took_meds_anti_tnf_alpha

Your Health Profile and Health History:

- Have you ever taken these medications? Anti-TNF alpha drugs (such as Humira, Remicade, Simponi, Cimzia, or Enbrel)
  coded as: cases: ‘yes’; controls: ‘no’; NAs: ‘I’m not sure

iqb.took_meds_asthma_or_lung

Your Health Profile and Health History:

- Have you ever taken these medications? Medications for asthma or another lung condition, such as COPD (Chronic Obstructive Pulmonary Disease)

coded as: cases: "Yes" ; controls: "No" ; NAs: "I'm not sure"

iqb.took_meds_prednisone_or_steroid

Your Health Profile and Health History:

- Have you ever taken these medications?"/"Prednisone or other steroids"
  Responses coded as: cases: "Yes" ; controls: "No" ; NAs: "I'm not sure"

male_pattern_grade_survival

All subjects are male, and participants provided responses to the

Hair Loss in Men and Women survey:

- Please choose the image that best captures your hair's pattern and density. If your head is shaved, please answer for how your hair looks when grown out. If none of these images are similar to your hair's pattern and density, choose none of the above. (Images a to s, corresponding to Hamilton scale)
- Have you experienced hair loss or thinning? (Yes, No, I'm not sure, Decline to state)
- How old were you when you first started to notice hair loss? (Under 18, 18-24, 25-29, 30-34, 35-39, 40-44, 45-49, 50-54, 55-59,

60-64, 65-69, 70-74, 75-79, 80 or older, I'm not sure)

Cases reported having experienced hair loss or thinning, and current hair loss of Hamilton grade higher than II. Controls are at least 40 years old, and report not having experienced hair loss or thinning, and at most Hamilton grade II. Assuming no risk for age <15.

male_pattern_meta

The male pattern baldness phenotype was defined for use in a recent meta-analysis (Li _et al._, _PLoS Genet_ 8: e1002746, 2012). All subjects are male, and participants provided responses to the

Hair Loss in Men and Women survey:

- Please choose the image that best captures your hair's pattern and density. If your head is shaved, please answer for how your hair looks when grown out. If none of these images are similar to your hair's pattern and density, choose none of the above. (Images a to s, corresponding to Hamilton scale)
- Have you experienced hair loss or thinning? (Yes, No, I'm not sure, Decline to state)
- How old were you when you first started to notice hair loss? (Under 18, 18-24, 25-29, 30-34, 35-39, 40-44, 45-49, 50-54, 55-59,
60-64, 65-69, 70-74, 75-79, 80 or older, I'm not sure)

Cases reported having experienced hair loss or thinning, with onset before age 40, and current hair loss of Hamilton grade III or higher. Controls are at least 30 years old, and report not having experienced hair loss or thinning, and at most Hamilton grade I, or if age 50 or older, Hamilton grade II.

nexium_any_problems

Cases are individuals who answered 'yes' to any of the following side effects or conditions; controls are individuals who answered 'no' to one of the following side effects or conditions, and did not provide any 'yes' answers.

Proton Pump Inhibitors Survey:

- Have you experienced any of these side effects from taking esomeprazole (Nexium)? Please only report side effects you believe may have been caused by esomeprazole."
  - Abdominal pain
  - Diarrhea
  - Dizziness
  - Headaches
  - Muscle pain or weakness
  - Nausea
  - Other Side effect

- Were you diagnosed with any of these conditions while taking esomeprazole (Nexium)? Please note: Later questions will ask about whether these problems were related to your use of esomeprazole."
  - Clostridium difficile infection
  - Inflammation of the kidneys
  - Bacterial pneumonia"

- Did you experience any of these side effects while taking esomeprazole (Nexium)? Please only report side effects you believe may have been caused by esomeprazole.
  - Abdominal pain
  - Diarrhea
  - Dizziness
  - Headaches
  - Muscle pain or weakness
  - Nausea

- Other Side effect

nsaid_efficacy

nsaid_efficacy is a binary trait to look at efficacy of non-steroidal anti-inflammatory drugs (nsaid: aspirin, ibuprofen, aleve) for relieving pain. The data comes from:

<u>Common medications survey</u>

- How much does aspirin relieve your pain?

(``It relieves my pain a great deal'', ``It relieves my pain some'',
``It relieves my pain a little'', ``It does not relieve my pain'',
``I'm not sure'')

- How much does ibuprofen relieve your pain?

(``It relieves my pain a great deal'', ``It relieves my pain some'',
``It relieves my pain a little'', ``It does not relieve my pain'',
``I'm not sure'')

- How much does naproxen (Aleve) relieve your pain?

(``It relieves my pain a great deal'', ``It relieves my pain some'',
``It relieves my pain a little'', ``It does not relieve my pain'',
``I'm not sure'')

Combined efficacy from three nsaid drugs: aspirin, ibuprofen, naproxen.
Cases are individuals who are cases for something but not controls for anything.
Controls are individuals who are controls for something but not cases for anything.
For each drug, cases are defined as answering ``It relieves my pain a little'' or
``It does not relieve my pain'' and controls are defined as answering ``It relieves my pain some'' or ``It relieves my pain a great deal''.

tonsillectomy

Data on ``tonsillectomy'' comes from two sources:

<u>Your Medical History survey:</u>

- Have you ever had any of the following surgeries? (Tonsillectomy)

<u>Research snippet:</u>

- Have you had your tonsils removed?

Cases gave a positive response to either question, and controls gave a negative response to either question. Individuals with discordant responses were excluded.
